# Autologous hinge-masked anti-TNFα antibody to mitigate immunogenicity, antigen sink effects, and anti-idiotypic antibody interference

**DOI:** 10.1186/s13036-026-00741-x

**Published:** 2026-08-01

**Authors:** Bo-Cheng Huang, Yun-Chi Lu, Chih-Hung Chuang, Shih-Ting Hong, Kai-Wen Ho, Yi-An Cheng, Tzu-Yi Liao, En-Shuo Liu, Jun-min Liao, Chiao-Yun Chen, Tian-Lu Cheng, I-Ju Chen, Wen-Wei Lin

**Affiliations:** 1 Drug Development and Value Creation Research Center, Kaohsiung Medical University, Kaohsiung, Taiwan; 2 Department of Medical Laboratory Science and Biotechnology, Kaohsiung Medical University, Kaohsiung, Taiwan; 3 PrecisemAb Biotech Co., Ltd., Taipei, Taiwan; 4 Graduate Institute of Medicine, College of Medicine, Kaohsiung Medical University, Kaohsiung, Taiwan; 5 Department of Biomedical Science and Environmental Biology, Kaohsiung Medical University, Kaohsiung, Taiwan; 6 Department of Medical Imaging, Kaohsiung Medical University Hospital, Kaohsiung, Taiwan; 7 School of Post-Baccalaureate Medicine, College of Medicine, Kaohsiung Medical University, Kaohsiung, Taiwan; 8 School of Medicine, I-Shou University, Kaohsiung, Taiwan; 9 Department of Laboratory Medicine, Post Baccalaureate Medicine, College of Medicine, Kaohsiung Medical University, Kaohsiung, Taiwan; 10 Department of Medical Research, Kaohsiung Medical University Hospital, Kaohsiung, Taiwan

**Keywords:** Anti-TNFα antibody, Anti-idiotypic antibody, Immunogenicity

## Abstract

**Background:**

Monoclonal antibody (mAb) therapies are limited by factors affecting their pharmacokinetics (PK), such as anti-idiotypic antibodies (anti-ID Abs) and the antigen sink effect. To address this, we developed six pro-anti-TNFα Abs (pro-Infliximab) incorporating unique hinge regions (“Ab locks”) from different immunoglobulin subtypes linked via protease-sensitive peptides. The steric shielding formed by the endogenous hinge’s disulfide bonds provides a modular design that can be applied to various Ab drugs with minimal structural modification. Compared to other spatial hindrance strategies, this Ab lock approach is anticipated to offer a favorable immunogenicity profile.

**Results:**

Among the variants, The IgG1 hinge exhibited the most robust masking capability among the evaluated domains, resulting in a 128-fold reduction in Infliximab’s antigen binding. IgG1 pro-Infliximab demonstrated low immunogenicity after repeated dosing and maintained stable PK in both naïve and immunized mice, retaining 74.2% serum levels one hour after TNFα challenge. It also reduced anti-ID Ab binding by 87.3-fold, while showing a trend toward decreasing Ab-mediated drug clearance in these treated mice.

**Conclusions:**

This IgG1 Ab lock strategy provides binding-level proof of concept for several pro-Abs, offering the potential to mitigate immunogenicity. It blocked systemic hTNFα in an acute target challenge model and reduced clearance to preserve pro-Infliximab PK, ultimately helping to enhance patient quality of life.

**Supplementary Information:**

The online version contains supplementary material available at https://doi.org/10.1186/s13036-026-00741-x.

## Background

Monoclonal antibodies (mAbs) serve as an effective biological agent for specifically neutralizing target antigens and inhibiting the progression of certain diseases (e.g., infectious diseases [1], autoimmune diseases [2] and malignancies [3]). This study aims to mitigate the antigen sink effect and anti-idiotypic antibody (anti-ID Ab) binding by developing a novel hinge-masked pro-antibody (pro-Ab) strategy to prolong the pharmacokinetics (PK) of mAbs. The PK properties of mAbs significantly contribute to their efficacy during Ab therapy [4]. However, several factors, such as anti-ID Ab and target-mediated clearance (the “antigen sink effect”), affect PK behavior, including serum concentration, half-life, and function of mAbs. These factors limit the clinical application of mAbs [4, 5]. For example, Klause et al. indicated that 54% of patients with rheumatoid arthritis (RA) who initiated treatment with anti-tumor necrosis factor alpha (TNFα) Ab (i.e., Infliximab) had anti-Infliximab idiotypic Ab after 6 weeks and the presence of anti-Infliximab idiotypic Ab increased the risk of adverse infusion reaction, shortened the half-life and decreased the therapeutic efficacy of Infliximab as compared with patients without anti-Infliximab idiotypic Ab [6]. Anti-CD47 Ab blocks the CD47-signal-regulatory protein alpha (SIRPα) axis to enhance macrophage-mediated phagocytosis of tumor cells [7]. However, because CD47 is ubiquitously expressed in normal tissues [8, 9], it creates a strong antigen sink effect that accelerates drug clearance and compromises therapeutic efficacy in murine models [10]. Neuropilin-1 (NRP1), a vascular endothelial growth factor (VEGF) receptor involved in tumor angiogenesis, is widely expressed in normal tissues such as the liver, lung, and pancreas [11, 12]. While the anti-NRP1 human MNRP1685A showed efficacy in xenograft models [13], its 7.6-fold faster serum clearance in tumor-bearing mice suggests that higher clinical doses are necessary to achieve therapeutic tumor exposure [14]. Thus, developing a strategy that avoids the interference of anti-ID Abs or the antigen sink effect in vivo is important for achieving more effective therapeutic efficacy with Ab drugs. Our Ab lock platform is designed to address these pharmacokinetic challenges and thereby enhance therapeutic efficacy.

Several strategies have been developed for preventing the formation of anti-ID Abs in host B cells or the antigen sink effect caused by antigen-repressing normal tissue. For instance, Vermeire et al. demonstrated that concomitant use of immunosuppressive agents such as methotrexate (MTX) in anti-TNFα Ab (i.e., Infliximab)-treated Crohn’s disease patients can efficiently reduce the incidence of anti-Infliximab idiotypic Ab from 73% to 46% (*P* < 0.001) and preserving higher Infliximab serum levels [15]. However, concurrent treatment of immunosuppressive agents leads to an increased relative risk of serious opportunistic bacterial and fungal infections and the development of lymphoma [16–18]. Kishimoto and Zhang’s groups suggested that combining rapamycin, which is a macrolide compound with both immunomodulatory and anti-tumor activity [19], with Adalimumab (anti-TNFα Ab) can significantly prevent anti-Adalimumab Ab formation [20, 21]. Nevertheless, the dosage of rapamycin required for suppressing immune response was toxic in a preclinical model [22]. Bauss and colleagues even generated a new form of pseudomonas exotoxin-conjugated anti-mesothelin Ab (immunotoxin), which has eliminated multiple B cell epitopes by genetic deletion or point mutation, for escaping B cell recognition and following humoral immune responses [23]. However, it has not been possible to identify and remove all B cell epitopes from mutant immunotoxin and the strategy may probably create new epitopes for the human adaptive immune system [24]. These studies reveal that it is hard to completely avoid the immunogenicity of Ab drugs without adverse effects. Moreover, the antigen sink effect is another obstacle to Ab therapy. In order to prevent the depletion of Ab drugs by the antigen sink effect during disease treatment, Abs designed specifically against disease-specific antigens were generated for specific targeted therapy. Reilly et al. developed ABT-806, an anti-EGFR Ab targeting the EGFRvIII mutant found in 25–30% of glioblastoma patients [25]. Compared to cetuximab, ABT-806 showed superior anti-tumor activity with reduced antigen sink effects and minimized on-target toxicity to normal epithelial cells [26]. However, tumor-specific antigens (TSAs) are not easy to discover and do not even emerge in some types of malignancies. Based on the above, current approaches to preventing anti-ID Ab interference in Ab therapy face safety concerns and risks of genetic mutations, while strategies to address the antigen sink effect encounter implementation challenges in identifying suitable TSAs. To overcome these limitations, we previously developed a spatial hindrance strategy employing an autologous Ab lock derived from the immunoglobulin G hinge domain that masks the antigen-binding site of Ab drugs via a matrix metalloproteinase (MMP)-2/9-cleavable peptide linker, thereby enabling localized TNFα neutralization in inflamed tissues and minimizing systemic toxicity. Previous studies have demonstrated that in an RA mouse model, pro-Infliximab undergoes selective, MMP-2/9-mediated cleavage specifically within inflamed tissues, demonstrating a disease-site activation profile. Notably, pro-Infliximab exhibited therapeutic efficacy comparable to parental Infliximab, while attenuating the adverse effects associated with systemic administration [27]. However, whether the spatial hindrance Ab lock can avoid the interference of anti-ID Ab and the antigen sink effect and further prolong the serum half-life of Ab drug in vivo are still unknown.

**Fig. 1:**
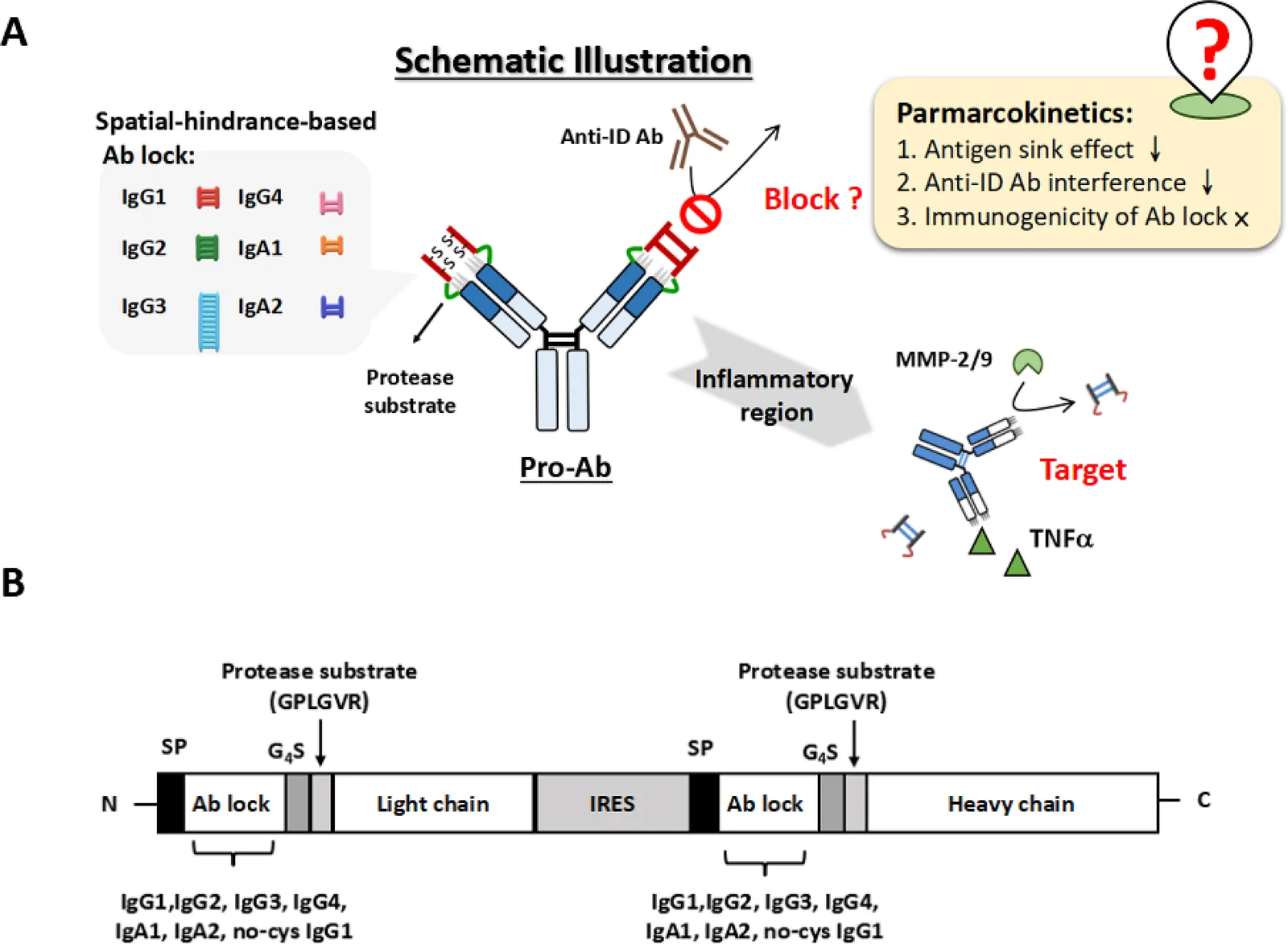
Proposed mechanism and schematic design of the autologous hinge-masked anti-TNFα antibody. (**A**) Pro-Abs were developed using various hinge domains (IgG1, IgG2, IgG3, IgG4, IgA1, and IgA2) as proposed Ab-locks. These are designed to cover the antigen-binding site by linking with a disease-specific protease substrate. Utilizing the endogenous disulfide of the Ab hinge, this spatial-hindrance-based Ab-lock is hypothesized to inhibit antigen binding at non-target sites, thereby potentially avoiding interference by anti-ID Abs or the antigen-sink effect in circulation to extend serum half-life. In this model, upon overexpression of MMP-2/9 at the inflammatory site, the protease cleaves the substrate to release the Ab-lock, theoretically allowing the pro-Ab to specifically activate and neutralize the antigen at the disease site. (**B**) DNA constructs for pro-Infliximab encode a signal peptide (SP), the hinge domain (IgG1, IgG2, IgG3, IgG4, IgA1 or IgA2), G4S flexible linker (GGGGS), protease substrate, light chain of Ab, internal ribosomal entry sequence (IRES), signal peptide (SP), the hinge domain, the G4S flexible linker (GGGGS), protease substrate, heavy chain of Ab

To achieve precise spatial hindrance for balancing masking efficiency with activation ability, and to verify the versatility of our platform across diverse antibody scaffolds, this study developed six pro-anti-TNFα antibodies (pro-Infliximabs) incorporating unique hinge regions (Ab-locks) derived from different immunoglobulin subtypes, including IgG1, IgG2, IgG3, IgG4, IgA1, and IgA2, linked via MMP-2/9 substrate peptides **(**Fig. 1A**)**. We first evaluated the masking efficiency of pro-Infliximabs on TNFα binding using enzyme-linked immunosorbent assay (ELISA). We then assessed the immunogenicity and clearance effects of Ab locks in immunocompetent mice through repeated dosing, including analysis of endogenous anti-Ab lock antibody responses. Furthermore, we investigated antigen sink effects and anti-ID Ab interference in vivo using systemic human TNFα (hTNFα) administration and anti-ID Ab-treated C57BL/6 mice. Finally, we demonstrated the potentially generalizable applicability of the Ab lock by incorporating it into therapeutic Abs targeting CTLA-4, IL-1β, CD52, EGFR, and PD-1, followed by antigen-binding analysis. Through these studies, we aim to establish a broadly applicable Ab lock platform capable of reducing off-target clearance and enhancing the PK of Ab therapeutics.

## Methods

### Cells and animals

Expi293F cells (Thermo Fisher Scientific, Waltham, MA, USA) were cultured in Expi293 Expression Medium at 37 °C in a humidified atmosphere of 8% CO_2_. C57BL/6 and BALB/c mice were purchased from the National Laboratory Animal Center, Taipei, Taiwan. Animal experiments were carried out in accordance with institutional guidelines and approved by the Animal Care and Use Committee of Kaohsiung Medical University (IACUC:106271), Kaohsiung, Taiwan.

### Pro-Ab construction, expression, and purification

The complementary DNA coding for the heavy and light chains of Abs were cloned based on Infliximab through assembly PCR. Human IgG1, IgG2, IgG3, IgG4, IgA1, IgA2 and no-cys IgG1 hinge sequences [28–30] (Suppl. Table 1) were obtained from the National Center for Biotechnology Information. The DNA constructs for each pro-Infliximab were subcloned into the pcDNA3.4 vector at the 5’ or 3’ terminus using NheI and ClaI restriction enzymes. Sequence confirmation was performed by Genomics BioSci. & Tech. to ensure accuracy. The hinges were linked to the N-terminus of the light chain and heavy chain of Infliximab through a matrix metalloproteinase (MMP)-2/9 substrate peptide (GPLGVR) and flexible linker (GGGGS) to generate an Ab lock by DNA cloning, as illustrated in Fig. 1B. The no-cys IgG1 hinge-encoding sequences consisted of the same sequence of IgG1 Ab lock, but the cysteine residue of the IgG1 hinge was mutated to serine. Coiled-coil peptide sequences were designed by the VelA1 (GASTTVAQLEEKVKTLRAENYELKSEVQRLEEQVAQL) and VelB1 (GASTSVDELQAEVDQLEDENYALKTKVAQLRKKVEKL) [31, 32]. The same protocol was applied to the construction of other Abs or pro-Abs. All Ab or pro-Ab production was through the Expi293F Expression System (Thermo Fisher Scientific) and they were purified using Protein A-Sepharose (GE Healthcare, Milwaukee, WI, USA). Of these, the yield of the IgG1 hinge pro-Infliximab is approximately 1 mg from 90 mL of Expi293F cell supernatant. To analyze the molecular weight and purity of purified Inflixmab and pro-Infliximab, 3 µg of each Ab was separated by 10% Sodium Dodecyl Sulphate-Polyacrylamide Gel Electrophoresis (SDS-PAGE) and then stained with Coomassie brilliant blue.

### Comparison of the binding ability of the pro-Infliximab with or without MMP-2/9 treatment

To determine the binding ability (EC_50_) of the hinges (IgG1, IgG2, IgG4, IgA1, IgA2 and no-cys IgG1) pro-Infliximab and Infliximab, TNFα (appropriate concentration: 0.3 µg/mL, Recombinant hTNFα Protein, E. coli-derived, R&D Systems, Catalog No. 210-TA, Lot# DDHB0318091, Minneapolis, MN, USA) was coated onto 96-well plates and blocked with 5% skim milk in PBS. Infliximab or pro-Infliximab were incubated with or without 20 µg/mL of MMP-2/9 (type IV collagenase, source from Clostridium histolyticum, Sigma-Aldrich, Catalog No. C5138, Lot# SLBJ1067V, St. Louis, MO, USA) in Dulbecco’s Modified Eagle Medium (DMEM)/0.05% bovine serum albumin (BSA) for 1 h at 37 °C before the reaction was terminated by bovine calf serum (BCS). All the samples were added onto the plates at the given concentrations (500-0.001 nM) for 1 h at RT. After washing, the wells were incubated for 1 h at room temperature (RT) with horseradish peroxidase (HRP)-conjugated goat anti-human IgG Fc antibody (Jackson ImmunoResearch Laboratories, West Grove, PA, USA) to detect the samples, and detection was performed by the addition of ABTS containing 30% H_2_O_2_. The binding ability was quantified through absorbance detection at 405 nm. The pro-anti human CTLA-4 Ab, pro-anti human CD52 Ab, pro-anti-IL-1β Ab, pro-anti-EGFR Ab and pro-anti-PD1 Ab activity levels were determined using the same protocol.

### Evaluation of the binding ability of anti-ID Abs to pro-Infliximab

Anti-idiotypic (Id) Fab form (appropriate concentration: 0.3 µg/mL, HCA212, Bio-Rad Laboratories, Redmond, WA, USA) was coated onto 96-well plates and blocked with 5% skim milk in PBS. Infliximab or hinges (IgG1, IgG2, IgG4, IgA1, IgA2 and no-cys IgG1) pro-Infliximab spiked in DMEM/0.05% BSA were added at the given concentrations at RT for 1 h. After washing, the plate was sequentially incubated with HRP-conjugated Anti-Id whole Ab (HCA213P, Bio-Rad Laboratories, Redmond, WA, USA). The plates were washed with PBS, and the detection was performed by the addition of ABTS containing 30% H2O2. The binding ability was quantified through absorbance detection at 405 nm.

### Measurement of anti-Ab lock Ab concentration in mice immunized with different types of Ab lock Ab

Eight- to twelve-week-old female BALB/c mice (*n* = 4 per group, randomly assigned) were immunized via intraperitoneal (IP) injection with 250 µg/kg of murine Infliximab (mIFX), mouse IgG1 hinge-murine Infliximab (mHinge-mIFX), human IgG1 hinge-murine Infliximab (hHinge-mIFX), coiled-coil peptide murine Infliximab (Coil-mIFX), Infliximab (chimeric anti-TNFα Ab), or PBS in a volume of 100 µL on days 1, 3, 6, 13, 20, and 27. Simultaneously, whole blood was collected via the tail vein on days 0, 6, 13, 20, 27 and 41. The different immunogenicity of Ab locks in mouse serum was detected by ELISA. In order to examine the level of anti-Ab lock Ab in immunized mice, 0.5 µg/mL pro-Infliximab (chimeric form), including mHinge-Infliximab, hHinge-Infliximab and Coil-Infliximab as antigens were coated onto 96-well plates and blocked with 5% skim milk. The serum samples at dilutions of 300-fold and serially-diluted Infliximab standards (in duplicate) were added to separate plates (50 µL/well) at 37 °C for 1 h. After washing, the plate was sequentially incubated with HRP-goat anti-mouse IgG Fcγ-HRP (min × Human) (Jackson ImmunoResearch Laboratories, West Grove, PA, USA) for 1 h at RT, and detection was performed by the addition ABTS containing 30% H_2_O_2_. The binding ability was quantified through absorbance detection at 405 nm.

### Evaluation of the clearance level of different types of pro-Infliximab in naïve and pre-immunized mice

Naïve mice and mice (*n* = 4 per group, randomly assigned) immunized with mHinge-mIFX, hHinge-mIFX, or Coil-mIFX were IP injected with 2.5 mg/kg of His-tagged mHinge-mIFX, His-tagged hHinge-mIFX, His-tagged Coil-mIFX, or PBS in 100 µL, respectively. Then, whole blood was collected via the tail vein at 0.5, 4, 24,72 and 144 h post-inoculation of the Ab in the mice. Each Ab concentration in mice serum was detected by ELISA. Rabbit anti-His Ab (Bethyl Laboratories) was coated onto 96-well plates and blocked with 5% skim milk. The serum samples at dilutions of 600-fold and serially-diluted Infliximab standards (in duplicate) were added to separate plates (50 µL/well) at 37 °C for 1 h. After washing, the plate was sequentially incubated with HRP-Goat anti-mouse IgG (Fab’)2 Ab (Jackson ImmunoResearch Laboratories, West Grove, PA, USA) for 1 h at RT, and detection was performed by the addition ABTS containing 30% H_2_O_2_. The binding ability was quantified through absorbance detection at 405 nm.

### Evaluation of the clearance level of Infliximab and pro-Infliximab-treated mice with or without hTNFα administration

To understand whether the Ab lock could prevent the systemic hTNFα binding and reduce the target-mediated clearance of pro-Infliximab, eight- to twelve-week-old female C57BL/6 mice (*n* = 3 per group, randomly assigned) were intravenously (IV) injected with 1.2 mg/kg of pro-Infliximab or Infliximab in a volume of 100 µL. At 120 min post-Ab injection, mice were IV injected with or without 0.5 mg/kg hTNFα (Recombinant hTNFα Protein, E. coli-derived, R&D Systems, Catalog No. 210-TA, Lot# DDHB0318091, Minneapolis, MN, USA) in 100 µL. As a control, a separate group received 0.5 mg/kg hTNFα alone in 100 µL without Ab. Whole blood was collected via the tail vein at different time points. The Ab concentration in mouse serum was detected by ELISA. The 96-well plates were coated with 0.5 µg/mL of goat anti-human IgG Fcγ Ab (Jackson ImmunoResearch Laboratories, West Grove, PA, USA) at 4 °C overnight. After blocking the plates with 5% skim milk in PBS overnight for 2 h at 37 °C, the wells were washed 3 times with PBS-T (PBS, 0.05% Tween-20) and once with PBS. The mouse serum samples at dilutions of 25- and 50-fold and serially-diluted Infliximab standards (in duplicate) were added to separate plates (50 µL/well) at 37 °C for 1 h. Unbound Abs were removed by washing the plates three times with PBS. HRP-goat anti-human IgG Fab Ab (Jackson ImmunoResearch Laboratories, West Grove, PA, USA) were added to the well, then the plates were washed with PBS three times. The bound Ab was measured by adding 150 µL/well ABTS solution (30% H_2_O_2_ and 100 mM phosphate-citrate, pH = 4.0) for 30 min at room temperature. The absorbance was measured at 405 nm with blank subtraction. The relative concentrations of Infliximab and pro-Infliximab in mouse serum samples were calculated by comparison with Infliximab standard curves, respectively. To evaluate the hTNFα concentration in mouse serum, the 96-well plates were coated with etanercept (Enbrel) for 2 h at 37 °C and blocked with 5% skim milk. The plates were incubated with 25- and 50-fold diluted mouse serum samples and serially-diluted hTNFα standards (in duplicate) were added to separate plates (50 µL/well) at room temperature for 1 h. The plates were washed with PBST and PBS, and sequentially incubated with mouse anti-hTNFα Ab at room temperature for 1 h. After washing, each sample was incubated with HRP-goat anti-human IgG Fab Ab (Jackson ImmunoResearch Laboratories, West Grove, PA, USA) for 1 h. The detection was performed by the addition of ABTS containing 30% H_2_O_2_. The relative concentrations of hTNFα concentration in mouse serum samples were calculated by comparison with hTNFα standard curves, respectively.

### Comparison of the pro-Infliximab concentration in mice with or without anti-Infliximab idiotypic Ab treatment

Eight- to twelve-week-old female C57BL/6 mice (*n* = 2 per group, randomly assigned) were IV injected with Infliximab (0.5 mg/kg) or pro-Infliximab (0.54 mg/kg) in 100 µL. All Abs were constructed with His and Myc tags. Ninety minutes post-inoculation of the Abs, mice were IV injected with 0.5 mg/kg anti-Infliximab idiotypic Ab (HCA213, Bio-Rad Laboratories, Redmond, WA, USA) in 100 µL. Whole blood was collected via the tail vein at 15, 80, 120, 400 min and 24, 48, 72 h. Each Ab concentration in mouse serum was detected by ELISA. 6-His Tag polyclonal Ab (2.5 µg/mL, A190-114 A, Bethyl Laboratories) was coated onto 96-well plates and blocked with 5% skim milk. The serum samples at dilutions of 100 or 300-fold and serially-diluted Infliximab standards (in duplicate) were added to separate plates (50 µL/well) at 37 °C for 1 h. After washing, the plate was sequentially incubated with Myc tag mAb Biotin (2 µg/mL, MA1-21316-BTIN, Invitrogen) for 1 h at RT. Then streptavidin (SA)-HRP (Jackson ImmunoResearch Laboratories, West Grove, PA, USA) was added for 30 min at RT and detection was performed by the addition of ABTS containing 30% H_2_O_2_. The binding ability was quantified through absorbance detection at 405 nm.

### Statistical analysis

Data are presented as mean ± SEM or SD. The immunogenicity and PK of different types of Ab locks in mice and the clearance of pro-Infliximab in the TNFα administration in mice were analyzed with multiple t-tests. The half-life of pro-Infliximab and Infliximab treated with or without anti-Infliximab idiotypic Ab in mice was calculated by one-phase decay exponential regression. Statistical analysis was performed using the GraphPad Prism v.6 and data were considered significant at a *P* value of less than 0.05.

## Results

### Generation and characterization of pro-Infliximab

To achieve precise spatial hindrance for balancing masking efficiency with activation ability, and to verify the versatility of our platform across diverse antibody scaffolds, we selected six hinge domain candidates, immunoglobulin G1 (IgG1), IgG2, IgG3, IgG4, IgA1, and IgA2, to serve as Ab locks for pro-Ab development. These hinge domains were chosen because antibodies naturally occur in human serum, and the disulfide bonds formed by endogenous cysteines within the hinge can provide the necessary spatial hindrance to effectively interfere with the antibody’s antigen-binding capacity. We also generated a no-cys IgG1 hinge Ab lock control construct, consisting of the same sequence of IgG1 Ab lock, but in the control construct, the cysteine residue of IgG1 hinge was mutated to serine, which is the most similar to cysteine out of all the amino acids. SDS-PAGE analysis showed that the Infliximab and pro-Infliximab were composed of a heavy chain (HC) and light chain (LC) under reducing and non-reducing conditions (Suppl. Figure 1). In the case of IgG3 hinge pro-Infliximab, the yield was 4-fold less than the parental Ab, which led to difficulty preparing enough pro-Ab for the subsequent experiments. We surmise that the multiple disulfide bonds of the IgG3 hinge led to the difficulty in Ab production, as compared to other hinge domains. Therefore, in the subsequent experiments, we focused on evaluating the functional tests of IgG1, IgG2, IgG4, IgA1 and IgA2 pro-Infliximab.

### The Ab lock can efficiently reduce the antigen binding ability of pro-Infliximab

**Fig. 2:**
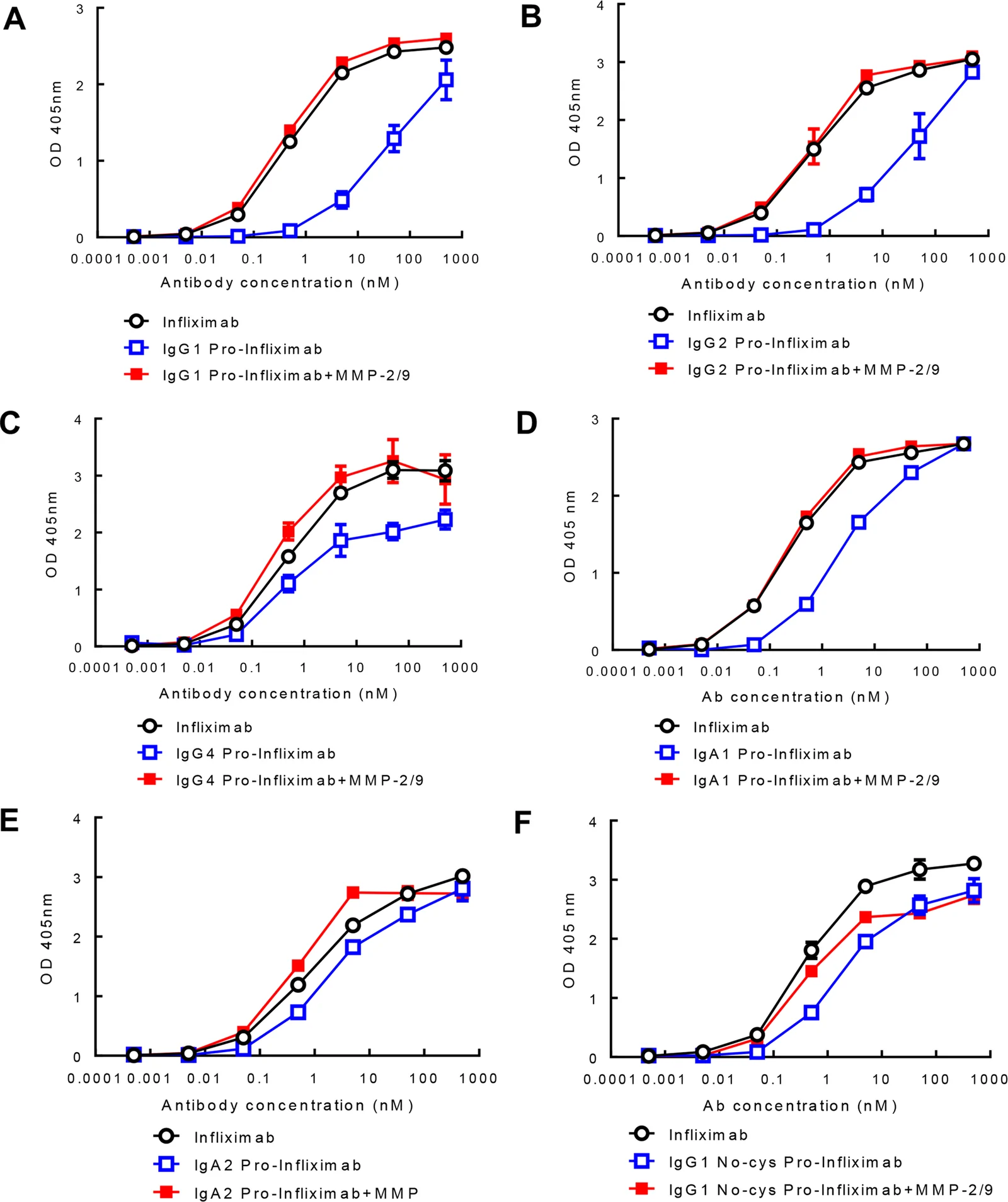
Inhibition of the binding ability of Infliximab by different hinge domains. (**A**) IgG1 pro-Infliximab, (**B**) IgG2 pro-Infliximab, (**C**) IgG4 pro-Infliximab, (**D**) IgA1 pro-Infliximab, (**E**) IgA2 pro-Infliximab, and (**F**) no-cys IgG1 pro-Infliximab were pre-incubated with MMP-2/9 to remove the hinge domain and restore the Ag-binding ability of Infliximab. Infliximab (○), pro-Infliximab (□) and MMP-2/9-activated pro-Infliximab (■) were then added to hTNFα-precoated 96-well plates in technical duplicate (*n* = 2) and detected using anti-human IgG Fcγ conjugated with horseradish peroxidase (HRP). Data are representative of 3 independent experiments

In order to investigate whether all the Ab locks could efficiently reduce the hTNFα (hTNFα)-binding ability, and completely restore the binding ability after MMP treatment, we incubated five pro-Infliximabs and Infliximab with MMP-2/9 and analyzed the antigen binding ability by hTNFα-based ELISA. As shown in Fig. 2, the IgG1, IgG2, IgG4, IgA1, and IgA2 pro-Infliximab showed 128.0-, 65.7-, 18.9-, 11.7-, 7.0-fold masking effect, respectively, after restoration by MMP-2/9 treatment. Among the hinge regions tested, IgG1 showed the highest masking efficiency, suggesting that it was the optimal candidate among the evaluated domains for pro-Infliximab (Table 1). Compared to other subclasses, IgG1 appears to be the most favorable backbone for masked antibody designs, likely because its moderate hinge length and proline-rich composition offer a superior balance of structural predictability and low conformational entropy. While the extreme flexibility of IgG3 may allow the mask to drift and the inherent instability of IgG4 could lead to inconsistent shielding, IgG1 seems to more reliably constrain the mask in close proximity to the CDRs. Furthermore, IgG1 avoids the excessive architectural complexity and rigidity associated with IgA, which potentially makes the engineering of precise, reversible activation more feasible than in a multi-domain dimeric framework. In addition, in Fig. 2F, the EC_50_ of no-cys IgG1 pro-Infliximab with or without protease treatment was 3.995 nM and 1.475 nM, respectively, and it only showed a 2.71-fold masking effect, which indicated that the disulfide bond provided by the cysteine residues in the hinge domain is critical to the masking ability of Ab lock to mAb. We demonstrated that the absence of the endogenous disulfide bond eliminates the steric masking effect of the pro-Infliximab Ab lock, thereby restoring antigen binding in Suppl. Figure 2 This was confirmed by predicting the molecular structures of both the IgG1 hinge pro-Infliximab and the no-cys IgG1 pro-Infliximab using Gaussian accelerated Molecular Dynamics (GaMD) simulations via AMBER software. As the next step, we further evaluated whether the autologous hinge domain-based Ab lock can reduce immunogenicity in vivo.

**Table 1:** Comparison of antigen-binding abilities among different hinge types

Hinge types	Pro-Ab EC_50_ (nM)	Pro-Ab + MMP EC_50_ (nM)	Masking fold
IgG1	56.33	0.44	128
IgG2	27.21	0.41	65.7
IgG4	4.7	0.25	18.9
IgA1	2.81	0.24	11.7
IgA2	3.35	0.48	7
IgG1 no-cys	3.99	1.48	2.7

Mean EC_50_ = median effective concentration

Masking fold = pro-Ab mean EC_50_/MMP-2/9-treated pro-Ab mean EC_50_

### The spatial-hindrance-based Ab lock can mask the antigen-binding ability of various clinical Ab drugs

In order to further investigate whether the Ab lock would be suitable for application to different kinds of therapeutic Ab drugs, we selected four candidate therapeutic Ab drugs in clinical use, anti-human cytotoxic T-lymphocyte-associated protein 4 (CTLA4), anti-human programmed cell death protein 1 (PD-1) Ab, anti-human interleukin 1 beta (IL-1β), anti-human EGFR (epidermal growth factor receptor), and anti-mouse CTLA4 Ab, and utilized the MMP-2/9 substrate peptide linker to construct pro-Abs using the same strategy as described above to generate pro-Abs. The masking effect and restoration ability of the pro-Abs were analyzed by performing individual antigen-based ELISA. The results demonstrated that the antigen binding EC_50_ of pro-anti-human CTLA4, pro-anti-human PD-1 Ab, pro-anti-human IL-1β, pro-anti-human EGFR, and pro-anti-mouse CTLA4 Ab were raised from 207.2 nM to 1.34 nM (154.6-fold masking effect), 29.32 nM to 0.62 nM (47.3-fold masking effect), 126 nM to 0.57 nM (221.1-fold masking effect), 141.5 nM to 0.6 nM (235.8-fold masking effect), 450.6 nM to 1.2 nM (375.5-fold masking effect) and 17.64 nM to 0.39 nM (45.2-fold masking effect), respectively, after restoration by MMP-2/9 treatment (Table 2). These results demonstrate that this spatial-hindrance-based Ab lock strategy provides binding-level proof of concept for several therapeutic Abs, effectively inhibiting the antigen-binding ability of Ab drugs with different antigen specificities.

**Table 2:** Binding-level proof of concept for several therapeutic Abs using the Ab lock strategy and their reactivation by protease

Antigen	Pro-Ab EC_50_ (nM)	Pro-Ab + MMP EC_50_ (nM)	Masking fold
Human TNFα	308.32	1.15	269.5
Human CTLA4	64.29	0.37	173.8
Human IL-1β	0.054	0.00042	128.6
Human CD52	9860	83.47	118.1
Human EGFR	28.94	0.57	50.8
Human PD-1	450.6	1.2	369.3

The Ab lock can inhibit the binding abilities of various types of Ab drugs, and the binding ability of pro-Abs can be revived by MMP treatment

Mean EC_50_ = median effective concentration

Masking fold = pro-Ab mean EC_50_/MMP-2/9-treated pro-Ab mean EC_50_

### The Ab lock can prevent the neutralizing effect of anti-ID Abs

**Fig. 3:**
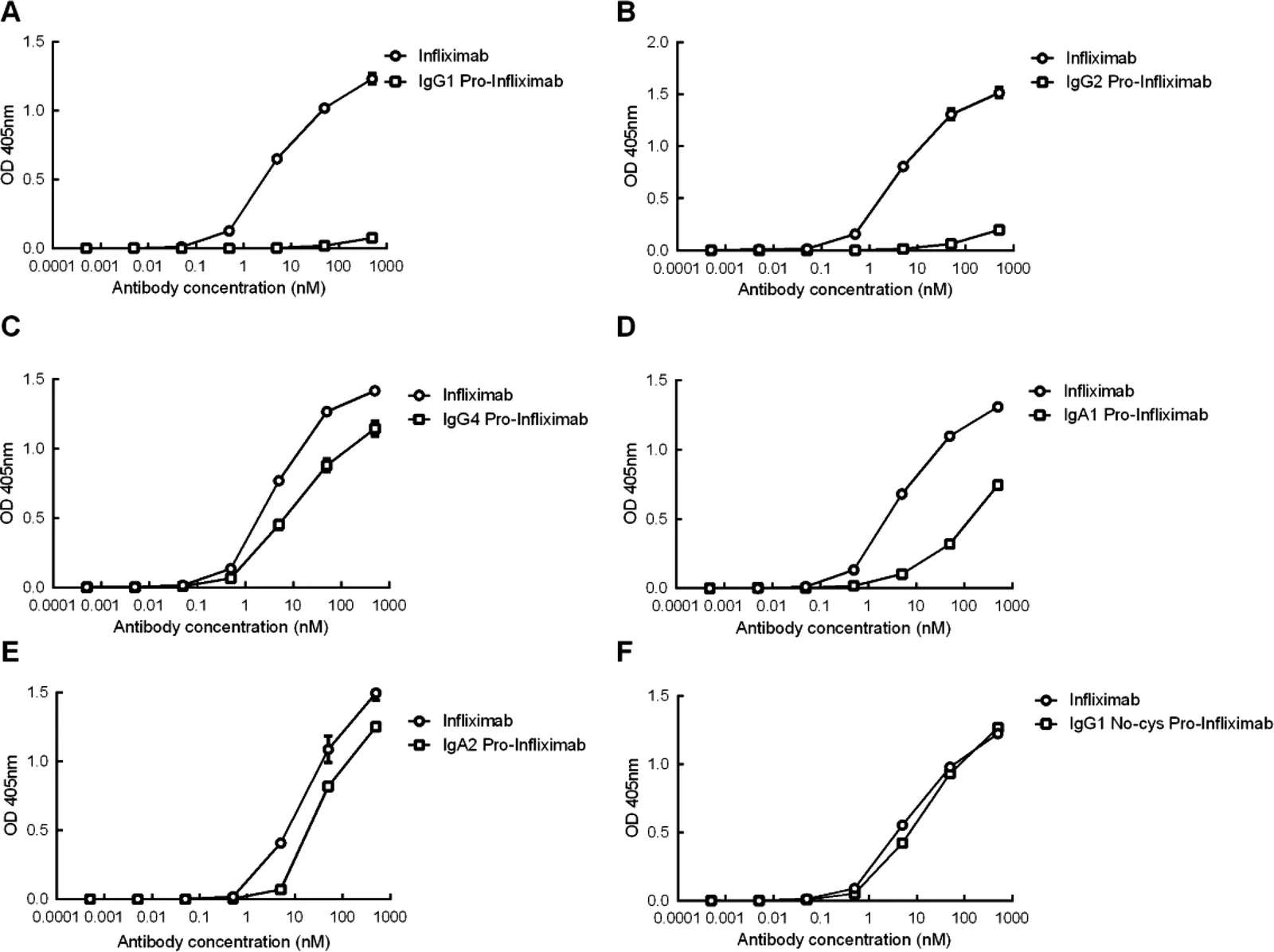
The Ab locks can prevent the binding of the anti-ID Ab to Infliximab. The anti-Infliximab-idiotypic Fab–coated 96-well plate was incubated with different concentrations of Infliximab, (**A**) IgG1 pro-Infliximab, (**B**) IgG2 pro-Infliximab, (**C**) IgG4 pro-Infliximab, (**D**) IgA1 pro-Infliximab, (**E**) IgA2 pro-Infliximab, (**F**) no-cys IgG1 pro-Infliximab in technical duplicate (*n* = 2), respectively. After removing unbound Infliximab or the pro-Infliximabs, we added anti-Infliximab-idiotypic Ab-conjugated horseradish peroxidase (HRP) to examine the binding ability of anti-Infliximab idiotypic Ab to Infliximab (○) and pro-Infliximab (□) by ELISA. Data are representative of 3 independent experiments. The values are mean ± SEM. Error bar: standard error of twice determinations. Ab, antibody; HRP, horseradish peroxidase; MMP, matrix metalloproteinase

High levels of anti-ID Abs neutralize the antigen binding site of Ab drugs, thus neutralizing their activity and increasing the clearance effect after Ab drug-anti-ID Ab complex formation, thereby decreasing the therapeutic efficacy of Ab therapy [33, 34]. Therefore, preventing Ab drugs from being affected by the neutralizing effect of anti-ID Abs may decrease the clearance effect of Ab drugs. To confirm whether the Ab lock could provide spatial hindrance to prevent the binding of the anti-Infliximab idiotypic Abs to protect pro-Infliximab, we immobilized a neutralizing anti-Infliximab idiotypic Fab and then incubated it with different concentrations of the five kinds of pro-Infliximab and Infliximab. After that, we treated the sample with neutralizing anti-Infliximab idiotypic whole Ab to analyze the binding ability of anti-Infliximab idiotypic Abs to pro-Infliximab and Infliximab. As shown in Fig. 3, the anti-Infliximab idiotypic Ab binding ability of all the pro-Infliximab was lower than Infliximab. All of the pro-Infliximabs had lower anti-Infliximab idiotypic Ab binding activity as compared to the control Infliximab. The difference between the control Infliximab and IgG1, IgG2, IgG4, IgA1, IgA2 pro-Infliximab was 87.3, 87.1, 1.2, 26.1 and 1.4-fold, respectively. Among them, IgG1 pro-Infliximab possessed the weakest anti-Infliximab idiotypic Ab binding activity as compared to control Infliximab, which means IgG1 Ab lock has the greatest protection efficiency in preventing anti-Infliximab idiotypic Abs neutralization. In addition, as shown in Fig. 3F, the binding of anti-Infliximab idiotypic Ab to no-cys IgG1 pro-Infliximab and Infliximab were similar, which indicated that the Ab lock lost its masking ability to anti-Infliximab idiotypic Ab without cysteine. Together, these results suggest that the IgG1 Ab lock is the most appropriate inhibitory domain of pro-Infliximab as it notably masked the antigen binding ability of Infliximab and prevented the interference effect of the anti-Infliximab idiotypic Ab.

### Pro-Infliximab inefficiently induced antibody response against the Ab lock (i.e., the hinge region) in vivo

**Fig. 4:**
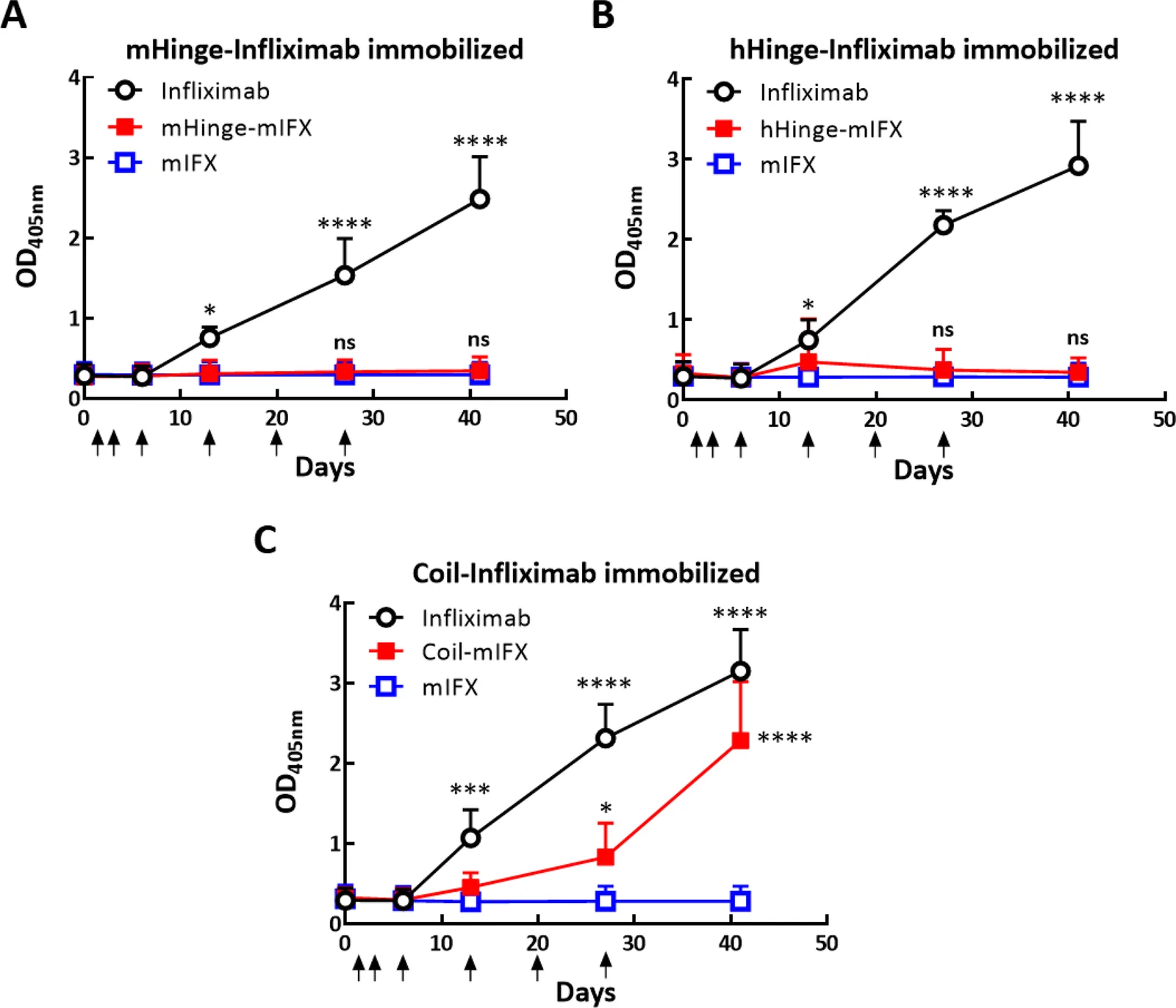
Immunogenicity of different types of Ab lock in BALB/c mice. Five micrograms of Infliximab, mIFX, mHinge-mIFX, hHinge-mIFX, or Coil-mIFX were injected intraperitoneally into BALB/c mice. Blood samples were collected at different time points to measure immune responses. (**A**) Immune response to mHinge-mIFX: serum Ab levels after injection of Infliximab (○), mHinge-mIFX (■), and mIFX (□) were measured by sandwich ELISA (*n* = 4). (**B**) Immune response to hHinge-mIFX: serum levels after injection of Infliximab (○), hHinge-mIFX (■), and mIFX (□) were measured (*n* = 4). (**C**) Immune response to Coil-mIFX: serum levels after injection of Infliximab (○), Coil-mIFX (■), and mIFX (□) were measured (*n* = 4). Black arrows show the days when injections were given. Data are represented as mean ± SD. Statistical analysis was performed by multiple t-tests. ****, *P* < 0.0001; ***, *P* < 0.001; *, *P* < 0.05; ns, not significant. Confidence intervals and effect sizes are shown in Suppl. Table 2 for comparison purposes

We investigated the immunogenicity of the Ab lock in BALB/c mice. We constructed the full murine Infliximab (mIFX) through replacing the human constant regions of Infliximab (chimeric anti-TNFα Ab) with mouse IgG1 constant regions, then different types of Ab lock (mHinge, mouse IgG1 hinge; hHinge, human IgG1 hinge; Coil, coiled-coil peptide) were linked to the N-terminus of the light chain and heavy chain of mIFX to generate mHinge-mIFX, hHinge-mIFX and Coil-mIFX pro-Abs, respectively (Suppl. Figure 3), of which the antigen-blocking and cleavage efficiency were assessed by ELISA in Suppl. Figure 4. BALB/c mice were repeatedly injected with Infliximab (chimeric), mIFX (full mouse), mHinge-mIFX, hHinge-mIFX and Coil-mIFX. The immunogenicities towards the different types of Ab lock were determined by sandwich ELISA which was immobilized with pro-Infliximab (chimeric form), mHinge-Infliximab, hHinge-Infliximab and Coil-Infliximab. As shown in Fig. 4, the Infliximab group was the positive control for the immunogenic assay, and the mIFX group was the negative control for the immunogenic assay. Significant differences between the Infliximab group and mIFX group were found on day 13 (*P* < 0.05), day 27 and day 41 (*P* < 0.0001) among the administration. Figure 4A and B present the immunogenic responses to mHinge-Infliximab and hHinge-Infliximab, respectively. There was no significant difference between the mHinge-mIFX or hHinge-mIFX group and mIFX group. Figure 3C shows the immunogenic responses to Coil-Infliximab, significant differences between the Coil-mIFX group and mIFX group on day 27 (*P* < 0.05) and day 41 (*P* < 0.0001). The comparative immunogenicity significant difference of different pro-Abs in mice over time are presented as Table 3. These results indicate that the coiled-coil peptide-based Ab lock induces a strong immune response in BALB/c mice, whereas neither mouse IgG1 nor human IgG1 hinges do. This suggests that the endogenous hinge Ab lock holds potential to mitigate the immunogenicity risks associated with Hinge domain-based pro-Abs.

**Table 3:** Comparative immunogenicity *P* values of different Ab locks in mice over time

	mHinge-Infliximab immobilized			hHinge-Infliximab immobilized			Coil-Infliximab immobilized		
	Day 13	27	41	Day 13	27	41	Day 13	27	41
Infliximab group vs. mIFX group	0.031, *	< 0.0001, ****	< 0.0001, ****	0.013, *	< 0.0001, ****	< 0.0001, ****	0.0002, ***	< 0.0001, ****	< 0.0001, ****
mHinge-mIFX vs. mIFX group	0.929, ns	0.758, ns	0.686, ns						
hHinge-mIFX vs. mIFX group				0.281, ns	0.627, ns	0.738, ns			
Coil-mIFX vs. mIFX group							0.413, ns	0.016, *	< 0.0001, ****

### The pharmacokinetics of pro-Abs in mice immunized with pro-Abs

**Fig. 5:**
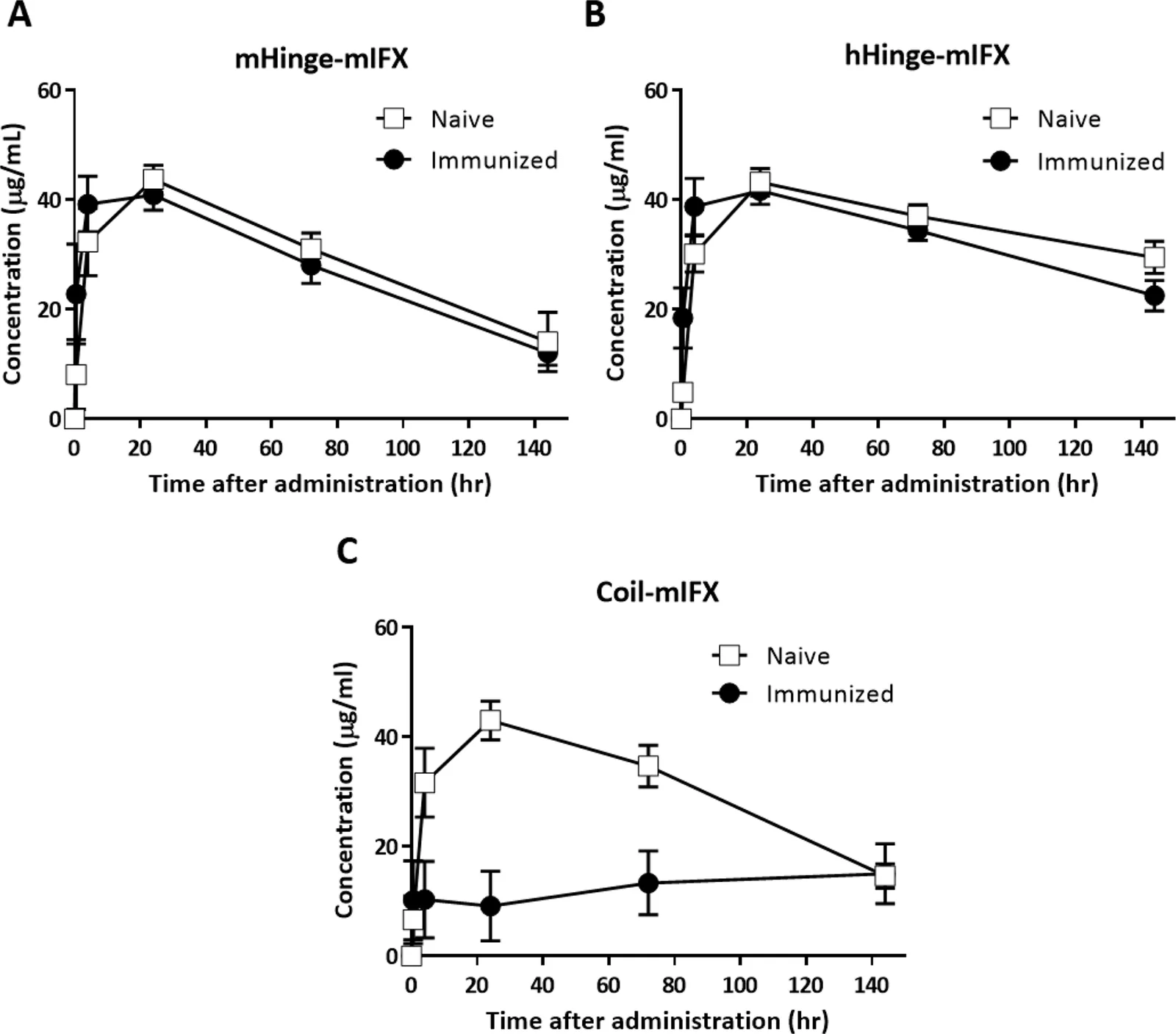
The pharmacokinetics of different types of pro-Abs in naïve mice and immunized mice. The PK of (**A**) mHinge-mIFX, (**B**) hHinge-mIFX and (**C**) Coil-mIFX in naïve BALB/c mice (white squares) and immunized BALB/c mice (black circles) were measured by an anti-His antibody-coated ELISA (*n* = 4). Data are represented as mean ± SEM. Statistical analysis was performed by multiple t-tests. Confidence intervals and effect sizes are shown in Suppl. Table 3 for comparison purposes

In order to demonstrate whether the low immunogenic Ab lock affects the PK of pro-Abs, BALB/c mice were divided into two groups, a naïve group and an immunized group (repeatedly injected with mHinge-mIFX, hHinge-mIFX and Coil-mIFX, respectively, to induce the immunogenic response). After confirming the immunogenic responses, mice were intraperitoneally injected with His-tagged mHinge-mIFX, hHinge-mIFX and Coil-mIFX, respectively, then serum samples were taken at various times and the concentration of pro-Abs was determined by sandwich ELISA with anti-His antibody as the capture agent. As shown in Fig. 5, the serum concentration trends of mHinge-mIFX (Fig. 5A) and hHinge-mIFX (Fig. 5B) were similar in both the naïve group and the immunized group. However, the serum concentration of Coil-mIFX (Fig. 5C) decreased faster in the immunized group than the naïve group. The difference between immunized mice and naïve mice was 4.3-fold at 24 h post administration of His-tagged Coil-mIFX. The comparative time-course of serum Ab concentrations in immunized and naïve mice is presented in Table 4. These results suggest that neither mHinge-mIFX nor hHinge-mIFX induced the anti-Hinge Ab response in the immunized group, thus, their PK properties were similar to the naïve group. In contrast, the Coil-mIFX induced the anti-Coil Ab response in the immunized group; therefore, its PK property was less than the naïve group.

**Table 4:** Time-course of serum antibody concentrations in immunized and naïve mice

	Time after administration (hr)					
	0.5	4	24	72	144	Statistical significance^4^
**mHinge-mIFX**						
naïve group (µg/mL)	8.14	32.32	43.78	31.05	14.08	
^1^immunized group (µg/mL)	22.82	39.25	40.92	28.08	12.08	ns
**hHinge-mIFX**						
naïve group (µg/mL)	4.85	30.13	43.21	37.02	29.48	
^2^immunized group (µg/mL)	18.41	38.76	41.69	34.41	22.50	ns
**Coil-mIFX**						
naïve group (µg/mL)	6.61	31.63	43.01	34.68	14.61	
^3^immunized group (µg/mL)	10.19	10.31	9.12	13.33	15.04	*P* < 0.0001

^1^repeatedly injected with mHinge-mIFX to induce the immunogenic response

^2^repeatedly injected with hHinge-mIFX to induce the immunogenic response

^3^repeatedly injected with Coil-mIFX to induce the immunogenic response

^4^statistical significance between the naïve and immunized groups at 24 h

### The Ab lock prolongs the pharmacokinetics of pro-Infliximab in the presence of hTNFα

**Fig. 6:**
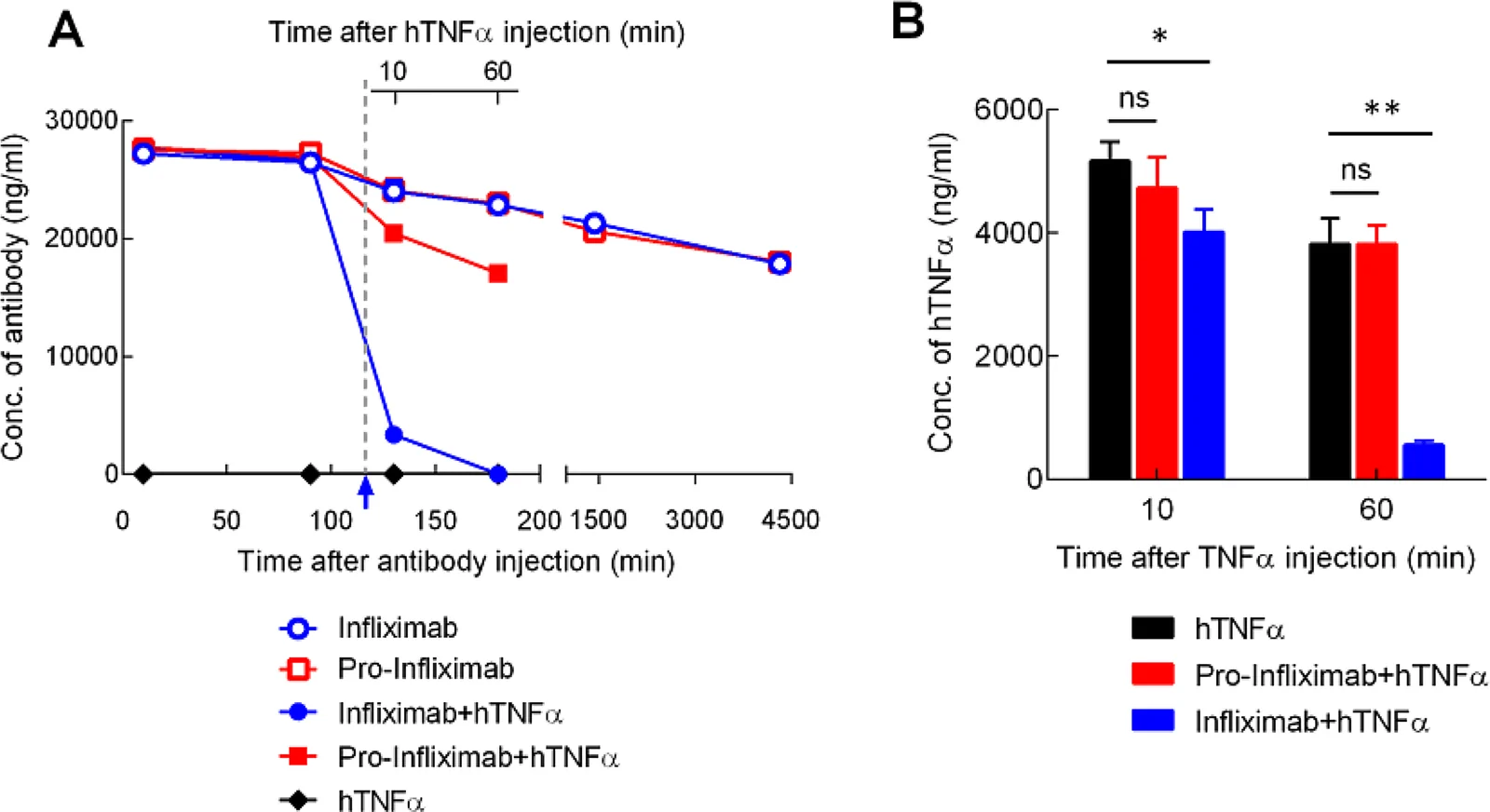
The Ab lock prevents systemic hTNFα binding and reduces the clearance of pro-Infliximab in an acute target challenge. Infliximab and pro-Infliximab were IV injected into C57BL/6 mice. After 120 min, the mice were intravenously injected with or without hTNFα (n = 3 per group). The hTNFα-only-treated mice were used as controls to evaluate the serum half-life of hTNFα in mice. Blood was harvested at different times. The serum concentrations of (**A**) Ab and (**B**) hTNFα were analyzed by quantitative ELISA. Day of hTNFα administration (blue arrow). Data are represented as mean ± SD. Statistical analysis was performed by comparing multiple t-tests. **, P < 0.01; *, P < 0.05; ns, not significant. Confidence intervals and effect sizes are shown in Suppl. Table 4 for comparison purposes

To understand whether the IgG1 Ab lock could provide spatial hindrance to prevent the systemic hTNFα binding and reduce the target-mediated clearance of pro-Infliximab, we established an acute target challenge model. In this setup, C57BL/6 mice (*n* = 3) were injected with Infliximab or pro-Infliximab, and further injected with an acute dose of hTNFα at 120 min post-administration of the Ab. To compare the PK of Infliximab and pro-Infliximab under this acute target challenge, the concentration of the Ab and hTNFα, respectively, was detected by sandwich ELISA. As shown in Fig. 6A, the titer of Infliximab with hTNFα in the blood was decreased 86% in mice as compared with Infliximab-treated mice without exogenous hTNFα. In contrast, the titer of pro-Infliximab with hTNFα only decreased 15% in mice as compared with pro-Infliximab-treated mice without exogenous hTNFα at 10 min post-administration of hTNFα. At 60 min post-inoculation of hTNFα, Infliximab with hTNFα was undetectable in the plasma, and the concentration of pro-Infliximab with hTNFα remained at 74.2% as compared with the pro-Infliximab-treated mice without hTNFα. These results indicated that the Ab lock can reduce the hTNFα clearance of pro-Infliximab in mice, as compared with Infliximab. In addition, as shown in Fig. 6B, the hTNFα concentration in PBS-, pro-Inflximab- and Inflximab-treated mice were decreased by 26%, 19.2% and 86.1%, respectively, from 10 to 60 min post-administration of hTNFα. Among the above, the hTNFα concentration showed no significant difference between pro-Infliximab and hTNFα-treated groups (*P* = 0.9965), a whereas there was significant difference between Infliximab and hTNFα-treated groups (*P* < 0.05), suggesting that pro-Infliximab but not Infliximab prevents the systemic hTNFα binding in healthy mice. The comparative effect of hTNFα on the clearance of Pro-Infliximab and Infliximab is presented in Table 5. These results indicated that pro-Infliximab prevents the target-mediated clearance and unwanted systemic-TNFα neutralization, which reduces the target-mediated clearance and prolongs the circulation time of pro-Infliximab.

**Table 5:** Effect of hTNFα on drug clearance of Pro-Infliximab and Infliximab

	without hTNFα		with hTNFα	
Time after Ab injection (min)	10	60	10	60
Infliximab (µg/mL)	24.01	22.85	3.36	0.00
Pro-Infliximab (µg/mL)	24.07	22.99	20.47	17.07

### The Ab lock appears to lessen the neutralizing effects of anti-Infliximab idiotype Ab, potentially preserving the pharmacokinetics of pro-Infliximab

**Fig. 7:**
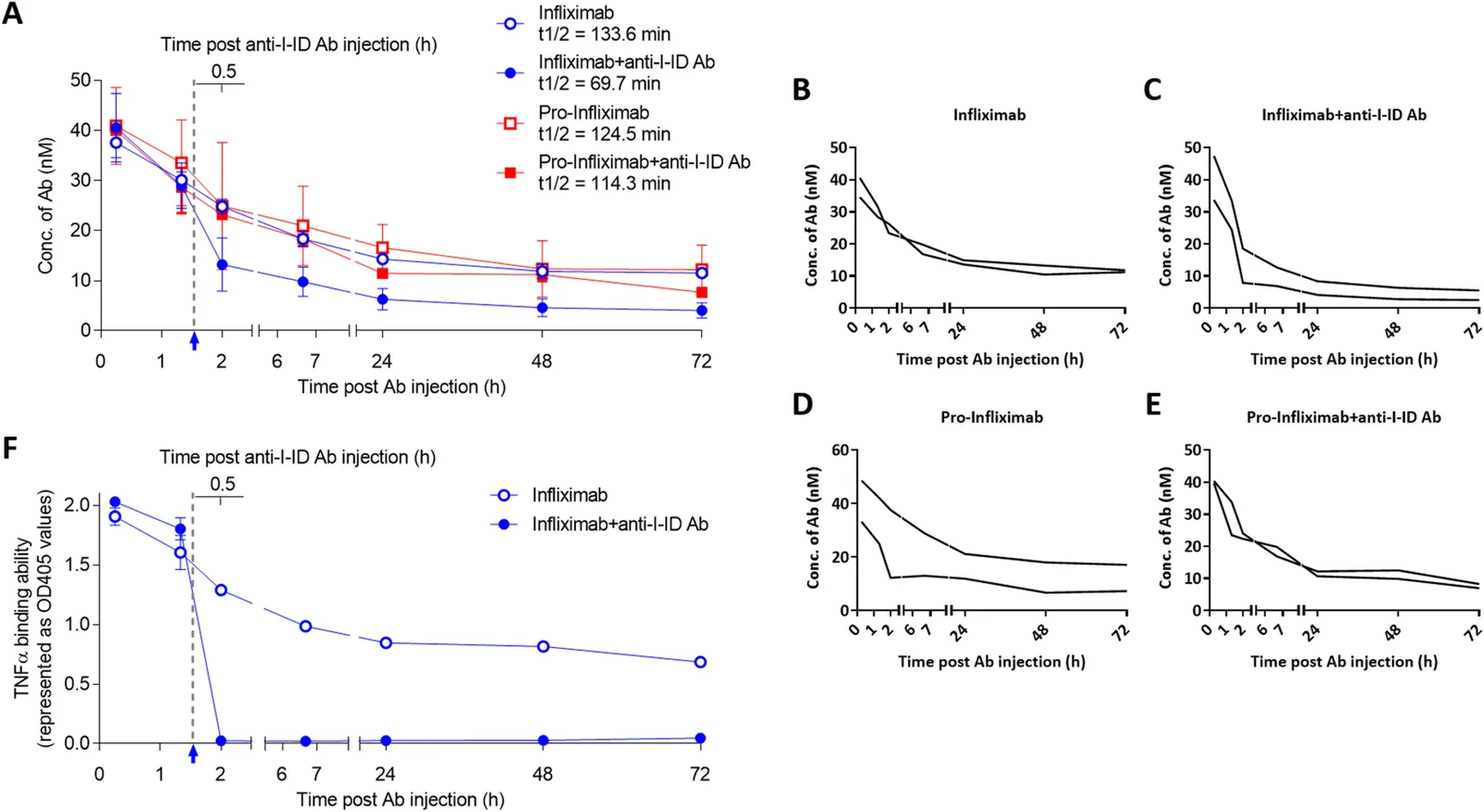
Preliminary trends of the Ab lock against anti-Infliximab idiotypic Ab interference and its antigen-blocking capability in blood. Infliximab and pro-Infliximab were IV injected into C57BL/6 mice. After 90 min, the mice were IV injected with or without anti-Infliximab idiotypic Ab (n = 2 per group). The blood was harvested at different times. The serum concentration of antibodies, represented as the (**A**) averaged data and (**B**-**E**) individual mouse data, along with the (**F**) hTNFα-binding ability, were analyzed by sandwich ELISA and hTNFα-based ELISA, respectively. Time of anti-Infliximab idiotypic Ab administration (blue arrow). Data are represented as mean ± SEM. Datapoints were fitted using a one-phase decay exponential regression, which allowed for the derivation of the half-life of Infliximab and pro-Infliximab to be calculated in each case. Anti-I-ID Ab: anti-Infliximab idiotypic Ab.

We further confirmed whether the Ab lock prevents the neutralizing of anti-Infliximab idiotype Abs and reduces the clearance of pro-Infliximab. The C57BL/6 mice (*n* = 2) were intravenously (IV) injected with Infliximab and pro-Infliximab, and further IV injected with or without anti-Infliximab idiotypic Abs at 90 min post-inoculation of Infliximab and pro-Infliximab. The PK of Infliximab and pro-Infliximab with or without anti-Infliximab idiotypic Abs were examined by sandwich ELISA. As shown in the averaged data of Fig. 7A and the individual mouse data in Figs. 7B-E, the titer of Infliximab with anti-Infliximab idiotypic Ab in the blood was decreased by 46.7% in mice as compared with Infliximab-treated mice without anti-Infliximab idiotypic Ab, but the titer of pro-Infliximab with anti-Infliximab idiotypic Ab only decreased by 6.9% in mice as compared with pro-Infliximab-treated mice without anti-Infliximab idiotypic Ab at 30 min post-injection of anti-Infliximab idiotypic Ab. The half-life of Infliximab and pro-Infliximab was 133.6 and 124.5 min, respectively, compared to 69.7 and 114.3 h for Infliximab with anti-Infliximab idiotypic Ab and pro-Infliximab with anti-Infliximab idiotypic Ab, respectively, suggesting that the Ab lock of pro-Infliximab prevented the interference of anti-Infliximab idiotypic Ab in mice as compared with Infliximab. In addition to affecting the concentration of Infliximab, we further evaluated whether the anti-Infliximab idiotypic Ab can impair the function of Infliximab by hTNFα-based ELISA. As shown in Fig. 7F, the hTNFα-binding function of Inflximab in Inflximab+anti-Infliximab idiotypic Ab treated-mice was decreased by 97.6% compared with Infliximab-treated mice at 30 min post-inoculation of anti-Infliximab idiotypic Ab, which indicated that an anti-Infliximab idiotypic Ab can cause dysfunction of Infliximab. The Ab lock appears to lessen the neutralizing effects of anti-Infliximab idiotype Ab, potentially preserving the PK of pro-Infliximab.

## Discussion

We have successfully developed an autologous hinge as a low-immunogenic Ab lock to prevent the TNFα antigen sink effect in an acute target challenge, while reducing anti-ID Ab binding, thereby prolonging the PK of pro-Infliximab. The IgG1 hinge variant exhibited the strongest masking effect, reducing Infliximab’s antigen binding by 128-fold. It demonstrated low immunogenicity after repeated dosing and maintained stable PK in both naïve and immunized mice, with 74.2% serum levels retained one hour after TNFα challenge. Moreover, it reduced anti-ID Ab binding by 87.3-fold, while showing a trend toward decreasing Ab-mediated drug clearance in these treated mice. In this study, antigen-sink effects were evaluated via an acute target challenge model involving IV administration to capture the immediate systemic interactions relevant to clinical Infliximab use. In contrast, immunogenicity assays utilized the IP route to provide a stringent stress test for the platform. The fact that the Ab-lock maintained low immunogenicity even under these challenging administration conditions further validates its robust shielding efficacy. Through disulfide bond-based spatial hindrance, this Ab lock strategy can also be broadly applied to various therapeutic Abs. We expect that in the future, this Ab lock may address important unmet needs in antibody therapy by enabling the conversion of conventional Abs into pro-Abs. By alleviating the side effects caused by systemic neutralization of target antigen, and preventing the antigen sink effect to maintain higher drug concentration in the disease area, the Ab lock allows the development of Abs that target previously undruggable antigens (e.g., EGFR and HER2), and it may provide a means by which to reappropriate Abs that have failed in clinical trials due to safety issues. For patients suffering from an anti-ID Ab and cannot tolerate the original Ab drug anymore, the Ab lock may provide another option with high efficiency but low immunogenicity. The potentially generalizable Ab lock suggests a new strategy to improve the PK properties and safety of Ab drugs.

It is important to develop pro-Abs with a low immunogenic masking domain so that they can reduce unwanted immunogenic responses and clearance of the pro-Ab. Biological drugs (e.g., recombinant Ab, protein or fusion protein)-induced immunogenicity is commonly observed in clinical settings. It is caused by repeated administration of biological drugs during disease treatment [35–37]. Immunogenic responses to biological drugs can impact both safety and PK properties, which can impact the utility and efficacy of the drugs [38]. Several strategies have been attempted to design pro-Abs with exogenous peptides that can be used as masking domains of Abs. For example, masking domains developed from a bacterial display library were utilized to mask the antigen-binding site of anti-EGFR Ab drugs [39, 40]. However, the foreign peptides and mutated antigen fragments were exogenous, which may increase immunogenicity and induce unnecessary responses when repeatedly injected into patients, thus limiting their long-term use in humans. The coiled-coil peptides were derived from leucine zipper heterodimeric coiled-coil domains for masking the binding domains of anti-CD20, anti-HER2 or anti-mouse CD3 Abs to generate coiled-coil pro-Abs [41]. However, the relatively complex helix structure of the coiled-coil masking domains may increase the risk of immunogenicity during long-term systemic administration of coiled-coil pro-Abs. Céspedes and colleagues demonstrated that immunization of eight α-helical coiled-coil peptides in immunized mice resulted in the development of specific IgG Abs to α-helical coiled-coil fragments. Among the administration of the eight different peptides, four peptides decreased and became negative after the third immunization, suggesting the α-helical coiled coil peptide could elicit immunogenicity and accelerate protein clearance in mice [42]. Our endogenous Ab locks, which are hinge domains, are derived from the Abs themselves and therefore lead to low immunogenicity as compared to exogenous peptides. The in vivo immunogenicity results indicated that both the mouse IgG1 hinges and human IgG1 hinges would not induce immune-related anti-Ab lock Ab production even after multiple administration, but coiled-coil peptides because the Ab lock could induce immunogenic responses in mice, suggesting that the endogenous hinge Ab lock possesses the low immunogenicity characteristics (Fig. 4). More importantly, the half-life of the endogenous hinge pro-Ab was better than coiled coil pro-Ab, indicating the lower immunogenicity Ab lock could decrease the clearance of pro-Ab (Fig. 5). These promising results suggest that the autologous and low immunogenic hinge domains are an efficient masking approach from which pro-Abs could be developed for clinical settings and likely increase patients’ compliance to achieve a better therapeutic index. Future studies using MAPPS or T cell proliferation assays could further elucidate the specific T-cell epitopes shielded by the Ab-lock, complementing the systemic immunogenicity profile observed in our current murine model.

The presence of neutralizing anti-ID Ab is a major issue during Ab drug disease treatment in the clinic. The variability of the CDR loop of Ab drugs may create epitopes that attract the attention of B cells or other antigen-presenting cells (APCs) and further induce the generation of Abs that can specifically recognize the idiotypic epitope on antigen binding site of the Ab drug. Anti-ID Abs do not only neutralize the antigen binding function of the Ab drug, but also increase the clearance effect after Ab drug-anti-ID Ab complex formation, thereby decreasing the therapeutic efficacy of Ab therapy. For example, about 12% of rheumatoid arthritis (RA) patients who received Adalimumab (a fully human IgG1 monoclonal Ab against hTNFα) treatment tested positive for anti-ID Ab against Adalimumab (approximately 98% with neutralizing ability) [43–45]. This kind of anti-ID Ab may result in the formation of immune complexes and accelerate its clearance from the circulation, thereby reducing the half-life or directly blocking the binding ability of Adalimumab to its target reducing treatment efficacy. Saito-Yabe et al. also demonstrated that anti-ID Ab was developed at around 10 days after treating humanized anti-human Fas Ab (R-125224), which can target Fas-expressing immune cells and be used to treat RA, in a collagen-induced arthritis cynomolgus monkey model. The anti-Fas idiotypic Ab accelerated the clearance rate of anti-Fas Ab in plasma and significantly shortened the half-life of anti-Fas Ab (3.3-fold), thereby decreasing the therapeutic efficacy of the disease [46]. Therefore, it is important to develop a strategy that can prevent Ab drugs from being affected by interference from the neutralizing and clearance effect of anti-ID Ab. In this study, we suggested that the spatial hindrance Ab lock can efficiently block the binding ability of anti-Infliximab idiotypic Ab to pro-Infliximab (Fig. 3) and further maintain the PK properties of pro-Infliximab no matter whether it is used in exogenous anti-Infliximab idiotypic Ab-treated (Fig. 7) or endogenous anti-drug Ab mouse model (Fig. 5). These results indicate that the Ab lock strategy may serve as a potential therapeutic option for patients who have already developed Ab drug-specific anti-ID Abs. Nevertheless, additional structural and mechanistic studies, such as high-resolution epitope mapping, are required to fully clarify the underlying mechanisms and to assess the broader applicability of the anti-ID Ab effect.

The antigen sink effect (also known as target-mediated clearance) is another important factor affecting the PK of Ab drugs during systemic treatment. When an Ab drug targets a soluble antigen or membrane antigen, the Ab-antigen immune complex can be eliminated by several routes, such as proteolysis by the reticuloendothelial system (RES) in the liver, target-specific endocytosis by target cells or non-specific pino- or endocytosis by normal cells, and nonlinearly decrease Ab concentration in plasma [47]. For instance, Trang et al. indicated that Ab against CD3, which is a protein complex that is widely expressed on T lymphocytes and is involved in its activation, was undetectable in plasma 2 days after intravenous administration in immunocompetent BALB/c mice because of the antigen sink effect caused by circulating T cells [41]. In contrast, the non-binding isotype control Ab can maintain serum level by at least 30% at day 15 post-administration [41]. They further suggested that αVβ6-binding Ab-drug conjugate (ADC) (h15H3-gluc-MMAE) was cleared rapidly after systemic administration due to αVβ6 broadly expressed on epithelial cells in different organs [41]. These studies reveal that the antigen sink effect can strongly interfere with the PK property and potentially reduce the therapeutic effect of Ab drugs. In this study, we demonstrated that Ab lock-masking Infliximab (pro-Infliximab) can significantly reduce the elimination effect of the target antigen (TNFα) and maintain the serum half-life of pro-Infliximab in an acute hTNFα target challenge mouse model (Fig. 6), suggesting that the spatial hindrance-based Ab lock may sterically interfere with the binding ability of the pro-Ab to target the antigen and protect Ab drugs from nonlinear target-mediated clearance. We expect that the pro-Ab strategy may be applicable to the development of Ab drugs that target broadly expressed antigens in normal tissue (e.g., epithelial cell adhesion molecule (EpCAM)), increasing the selectivity of Ab drugs to the disease site and even avoiding unexpected on-target toxicity caused by Ab drugs during systemic administration.

The development of a potentially generalizable Ab lock to enhance the selectivity of Ab drugs is very important. In previous studies, antigen-binding fragments developed from a bacterial display library were utilized to mask the antigen-binding site of Ab drugs by affinity hindrance [39, 48, 49]. However, these affinity-based approaches may be restricted by the difficulty in finding the balance between the masking effect and restoring the function of the masking peptide. For example, the results related to CD71-Targeting Probody Drug Conjugate (PDC) developed by Cytomx and Abbvie showed the higher affinity mask can inhibit greatly CD71 binding than the lower affinity mask; however, the higher affinity mask is less efficacious than a lower affinity mask in the tumor-bearing mouse model, which indicated the higher affinity mask might not be able to be removed from Ab binding site and cause the probody drug conjugate to not be able to be restored to full function [50]. The time-consuming process of selecting the most suitable masking peptide for each individual Ab might slow down the development of affinity-based approaches, thus limiting their application. In this study, the Ab lock’s masking effect originated from the steric shield formed by endogenous disulfide bonds in the hinge. The different hinges would not have affinity with the Ab, and can be completely removed from the original Ab [27, 51]. The biological functions of pro-Infliximab and pro-Canakinumab were indeed completely restored after cleaving by MMP-2/9. In this study we confirmed that the Ab lock can be successfully applied to several monoclonal human Abs, such as anti-human CTLA4, anti-human IL-1β, anti-human CD52, anti-human EGFR, and anti-human PD1 Ab. We will further investigate its implications for each target, including ongoing work with pro-anti-CTLA-4 and pro-anti-PD-1 Abs in melanoma. However, due to the existence of a disulfide bond in the Ab lock, there might be heterogeneity during the reduction and re-oxidation steps in the cysteine-conjugated Ab-drug conjugate production causing difficulty in scaling up the chemistry, manufacture and control (CMC) process [52]. But other kinds of drug conjugation strategies like lysine-conjugated or site-specific conjugated will not have this limitation for the Ab lock [52]. Moving forward, we plan to integrate AI-assisted prediction with laboratory validation to optimize both antigen-blocking efficiency and manufacturability. Additionally, the diversity of protease expression patterns across different diseases and among individual patients may result in variable protease activation of the pro-Ab. As a next step, we will evaluate the efficiency of protease-mediated activation of the pro-Ab in frozen tumor specimens. We hope the Ab lock can provide a strategy to convert therapeutic Abs or ADCs into selectively reactive pro-Abs, thereby improving PK properties and reducing on-target toxicities during systemic administration.

## Conclusion

Our study provides proof-of-concept, primarily in mouse models, that Ab lock-based Abs can modulate pharmacokinetic challenges, including the antigen sink effect in an acute target challenge model and anti-ID Ab interference, thereby potentially preserving PK and mitigating immunogenicity risks. These findings successfully provide binding-level proof of concept for several therapeutic Abs using this IgG1 Ab lock strategy. The Ab lock has the following advantages: (1) It is the first Ab lock that uses the Ab’s own structure as a masking domain, which can avoid the production of anti-Ab lock Abs caused by drug administration to affect the clinical development; (2) It can prevent the antigen sink effect to greatly enhance the PK of Pro-Ab; (3) The Ab lock-based platform effectively prevents anti-ID Ab binding; (4) It could efficiently cover the CDR loop of the Ab to reduce the interference effect of anti-ID Ab as compared with original Ab and reduce the drug clearance due to neutralizing anti-drug Ab administration; (5) The steric shielding formed by disulfide bonds suggests that the Ab lock can be applied across a broad range of therapeutic Abs [53]. The Ab lock can be applied to numerous Ab drugs by changing the substrate linker for disease-associated proteases with specific diseases and the masking efficiency can be further improved by masking critical residues for antigen binding of Ab based on structural adjustment. Compared to similar strategies in this field, the Ab lock might have the lowest immunogenicity discovereds to date. Future work will focus on further validation in humanized models and clinical studies, including the evaluation of long-term immunogenicity and chronic dosing responses. Given the clinical shift from chimeric frameworks toward highly humanized and fully human scaffolds to minimize anti-drug antibody (ADA) responses, it is essential to explore how the Ab-lock can further synergize with these modern templates. We expect that this high-potential technology can be extended to various antibody-derived formats, such as antibody-drug conjugates (ADCs) and bispecific antibodies (BsAbs), thereby revolutionizing the development of biologics. By providing an additional layer of structural shielding, we hope the Ab-lock will significantly optimize current antibody drug behavior, accelerate clinical translation, and ultimately provide a more convenient and superior quality of life for patients.

## Supplementary Information

Below is the link to the electronic supplementary material.

Supplementary Material 1

## Data Availability

All data needed to evaluate the conclusions in the paper are present in the paper and/or the Supplementary Materials. Additional data related to this paper may be requested from the authors.
