# Supplementary material for: Autologous hinge-masked anti-TNFα antibody to mitigate immunogenicity, antigen sink effects, and anti-idiotypic antibody interference
Source: J Biol Eng. 2026 Aug 1;20:158. doi: 10.1186/s13036-026-00741-x (PMC13721414; doi:10.1186/s13036-026-00741-x)
Supplement: Supplementary file 1 [file 13036_2026_741_MOESM1_ESM.docx]

**Supplementary Table 1:**

Human IgG1, IgG2, IgG3, IgG4, IgA1, IgA2, no-cys IgG1 and mouse IgG1 hinge sequences

| **Hinge types** | **Numbers of disulfide bonds** | **Overall hinge lengths (aa)** | **Amino acid sequences** |
| --- | --- | --- | --- |
| IgG1 | 3 | 15 | EPKSCDKTHTCPPCP |
| IgG2 | 4 | 12 | ERKCCVECPPCP |
| IgG3 | 11 | 62 | ELKTPLGDTTHTCPRCPEPKSCDTPPPCPRCPEPKSCDTPPPCPRCPEPKSCDTPPPCPRCP |
| IgG4 | 2 | 12 | ESKYGPPCPSCP |
| IgA1 | 2 | 23 | PVPSTPPTPSPSTPPTPSPSCCH |
| IgA2 | 2 | 10 | PVPPPPPCCH |
| no-cys IgG1 | 0 | 15 | EPKSSDKTHTSPPSP |
| Mouse IgG1 | 4 | 13 | VPRDCGCKPCICT |

**References**
1. Liu H, May K. Disulfide bond structures of IgG molecules: structural variations, chemical modifications and possible impactsto stability and biological function. MAbs. 2012;4(1):17-23. doi:10.4161/mabs.4.1.18347
2. Woof J, Russell M. Structure and function relationships in IgA. Mucosal Immunol. 2011;4(6):590-597. doi:10.1038/mi.2011.39

**Supplementary Figure 1A.**

**Reducing SDS-PAGE of puriﬁed Infliximab, IgG1, IgG2, IgG3, IgG4, IgA1 or IgA2 pro-Infliximab, no-cys IgG1 pro-Infliximab.** The HC molecular weights of Infliximab, IgG1, IgG2, IgG3, IgG4, IgA1, and IgA2 pro-Infliximab was 54, 58, 57, 63, 57, 59 and 57 kDa, respectively; the LC molecular weights of Infliximab, IgG1, IgG2, IgG3, IgG4, IgA1, and IgA2 pro-Infliximab were 26, 29, 29, 35, 29, 31 and 29 kDa, respectively, which were the expected molecular weights of all the pro-Abs.

**Supplementary Figure 1B.**

**Non-reducing SDS-PAGE of puriﬁed Infliximab and IgG1 pro-Infliximab.** The non-reducing SDS-PAGE analysis demonstrated the high purity and low aggregation status of both Infliximab and IgG1 pro-Infliximab. Furthermore, SEC-HPLC analysis confirmed that the monomeric fractions of Infliximab and IgG1 pro-Infliximab were 98.42% and 94.65%, respectively.

**Supplementary Figure 2.**

**The role of disulfide bonds in Ab locks of pro-Infliximab.** Computer molecular dynamic simulation of IgG1 pro-Infliximab (A) and no-cys IgG1 pro-Infliximab (B). The CDRs of light chain (green) and heavy chain (purple) are additionally marked to distinguish them from the Fab structure (grey). The Ab lock structures of IgG1 hinge (A) and no-cys IgG1 hinge (B) are shown in blue. The disulfide bonds of IgG1 hinge (A) are shown in red.

**Supplementary Figure 3.**

(A) SDS-PAGE analysis of mouse Infliximab (mIFX) and its pro-antibody variants: mHinge-mIFX, Coil-mIFX, and hHinge-mIFX. Under reducing conditions, the heavy chain (HC) and light chain (LC) for each construct were observed at their expected molecular weights: mIFX (HC: 49.92 kDa, LC: 23.43 kDa), mHinge-mIFX (HC: 51.17 kDa, LC: 24.68 kDa), Coil-mIFX (HC: 61.17 kDa, LC: 34.68 kDa), and hHinge-mIFX (HC: 52.67 kDa, LC: 26.18 kDa). **(B)** SDS-PAGE analysis of human Infliximab (IFX) and its pro-antibody variants: mHinge-IFX, Coil-IFX, and hHinge-IFX. Under reducing conditions, the heavy chain (HC) and light chain (LC) for each construct were observed at their expected molecular weights: IFX (HC: 55.00 kDa, LC: 25.60 kDa), mHinge-IFX (HC: 56.25 kDa, LC: 26.85 kDa), Coil-IFX (HC: 66.25 kDa, LC: 36.85 kDa), and hHinge-IFX (HC: 58.00 kDa, LC: 29.40 kDa).

**Supplementary Figure 4.**

(A) **Comparison of antigen-blocking and cleavage efficiency of mHinge-mIFX and coil-mIFX.** To assess antigen-blocking and cleavage efficiency, ELISA compared the binding affinities of pro-antibodies toward TNFα. Relative to the parental mIFX (EC-30 = 0.1334), both mHinge-mIFX and Coil-mIFX showed robust masking in the absence of MMP-2/9 (EC-30 of 3.2421 and 6.999, respectively). Upon protease treatment, binding was restored to levels comparable to mIFX (EC-30 of 0.1452 and 0.2008), confirming efficient proteolytic activation. **(B) Comparison of antigen-blocking and cleavage efficiency of mHinge-IFX and Coil-IFX.** ELISA results demonstrated that mHinge-IFX and Coil-IFX were effectively masked (EC-30 = 14.86 and 11.09) compared to parental IFX (EC-30 = 0.2043). Upon MMP-2/9 treatment, binding affinities were restored to 0.2445 (72-fold) and 0.0833 (133-fold), respectively, yielding binding profiles comparable to the control.

**Supplementary Table 2A**

**Infliximab group vs. mIFX group (mHinge-Infliximab immobilized)**

| **Day** | ***P* value** | **Mean1** | **Mean2** | **Difference** | **CI Lower** | **CI Upper** | **Effect size**  **(Cohen’s d)** |
| --- | --- | --- | --- | --- | --- | --- | --- |
| 0 | 0.940822 | 0.299833 | 0.285 | 0.0148333 | -0.397881741 | 0.427548341 | 0.032830844 |
| 6 | 0.916965 | 0.291667 | 0.270833 | 0.0208333 | -0.391881741 | 0.433548341 | 0.046110766 |
| 13 | 0.0309647 | 0.296667 | 0.756167 | -0.4595 | -0.872215041 | -0.046784959 | -1.017020687 |
| 27 | 0.0001 | 0.293667 | 1.53575 | -1.24208 | -1.703508083 | -0.780651917 | -2.458895737 |
| 41 | 0.0001 | 0.294667 | 2.4855 | -2.19083 | -2.603545041 | -1.778114959 | -4.849008555 |

**Supplementary Table 2B
mHinge-mIFX vs. mIFX group (mHinge-Infliximab immobilized)**

| **Day** | ***P* value** | **Mean1** | **Mean2** | **Difference** | **CI Lower** | **CI Upper** | **Effect size**  **(Cohen’s d)** |
| --- | --- | --- | --- | --- | --- | --- | --- |
| 0 | 0.827 | 0.3 | 0.272 | 0.028 | -0.234 | 0.29 | 0.094 |
| 6 | 0.914 | 0.292 | 0.278 | 0.014 | -0.248 | 0.276 | 0.046 |
| 13 | 0.929 | 0.297 | 0.308 | -0.011 | -0.273 | 0.251 | -0.038 |
| 27 | 0.758 | 0.294 | 0.333 | -0.039 | -0.301 | 0.223 | -0.133 |
| 41 | 0.686 | 0.295 | 0.346 | -0.052 | -0.314 | 0.211 | -0.175 |

**Supplementary Table 2C
hHinge-mIFX vs. mIFX group (hHinge-Infliximab immobilized)**

| **Day** | ***P* value** | **Mean1** | **Mean2** | **Difference** | **CI Lower** | **CI Upper** | **Effect size**  **(Cohen’s d)** |
| --- | --- | --- | --- | --- | --- | --- | --- |
| 0 | 0.817986 | 0.294875 | 0.3355 | -0.040625 | -0.397987961 | 0.316737961 | -0.058041353 |
| 6 | 0.961579 | 0.28525 | 0.27675 | 0.00849999 | -0.348862971 | 0.365862951 | 0.012144023 |
| 13 | 0.280649 | 0.283375 | 0.475625 | -0.19225 | -0.549612961 | 0.165112961 | -0.274669539 |
| 27 | 0.627166 | 0.2885 | 0.374375 | -0.085875 | -0.443237961 | 0.271487961 | -0.12269049 |
| 41 | 0.7378 | 0.285875 | 0.345 | -0.059125 | -0.416487961 | 0.298237961 | -0.084472492 |

**Supplementary Table 2D
Coil-mIFX vs. mIFX group (Coil-Infliximab immobilized)**

| **Day** | ***P* value** | **Mean1** | **Mean2** | **Difference** | **CI Lower** | **CI Upper** | **Effect size**  **(Cohen’s d)** |
| --- | --- | --- | --- | --- | --- | --- | --- |
| 0 | 0.949906 | 0.312 | 0.32575 | -0.01375 | -0.457004814 | 0.429504814 | -0.015838094 |
| 6 | 0.967654 | 0.289375 | 0.29825 | -0.008875 | -0.452129814 | 0.434379814 | -0.01022277 |
| 13 | 0.412501 | 0.275375 | 0.45575 | -0.180375 | -0.623629814 | 0.262879814 | -0.207767001 |
| 27 | 0.0162419 | 0.28025 | 0.833 | -0.55275 | -0.996004814 | -0.109495186 | -0.636691393 |
| 41 | <0.0001 | 0.280625 | 2.2855 | -2.00487 | -2.448124814 | -1.561615186 | -2.309332381 |

**Supplementary Table 3A
mHinge-mIFX in naïve BALB/c mice and immunized BALB/c mice**

| **Time (hr)** | ***P* value** | **Mean1** | **Mean2** | **Difference** | **CI Lower** | **CI Upper** | **Effect size**  **(Cohen’s d)** |
| --- | --- | --- | --- | --- | --- | --- | --- |
| 0 | >0.9999 | 0 | 0 | 0 | -13.23313083 | 13.23313083 | 0 |
| 0.5 | 0.0306177 | 22.8231 | 8.13846 | 14.6846 | 1.451469172 | 27.91773083 | 0.516310307 |
| 4 | 0.295746 | 39.2462 | 32.3231 | 6.92308 | -6.310050828 | 20.15621083 | 0.243415385 |
| 24 | 0.662757 | 40.9154 | 43.7846 | -2.86923 | -16.10236083 | 10.36390083 | -0.100882082 |
| 72 | 0.651798 | 28.0846 | 31.0538 | -2.96923 | -16.20236083 | 10.26390083 | -0.104398081 |
| 144 | 0.760976 | 12.0846 | 14.0846 | -2 | -15.23313083 | 11.23313083 | -0.070319969 |

**Supplementary Table 3B**
**hHinge-mIFX in naïve BALB/c mice and immunized BALB/c mice**

| **Time (hr)** | ***P* value** | **Mean1** | **Mean2** | **Difference** | **CI Lower** | **CI Upper** | **Effect size**  **(Cohen’s d)** |
| --- | --- | --- | --- | --- | --- | --- | --- |
| 0 | >0.9999 | 0 | 0 | 0 | -8.499011844 | 8.499011844 | 0 |
| 0.5 | 0.00260591 | 18.4091 | 4.85065 | 13.5584 | 5.059388156 | 22.05741184 | 0.742251848 |
| 4 | 0.0467526 | 38.7597 | 30.1299 | 8.62987 | 0.130858156 | 17.12888184 | 0.472440477 |
| 24 | 0.719032 | 41.6883 | 43.2078 | -1.51948 | -10.01849184 | 6.979531844 | -0.083183623 |
| 72 | 0.53727 | 34.4091 | 37.0195 | -2.61039 | -11.10940184 | 5.888621844 | -0.142905269 |
| 144 | 0.104445 | 22.5 | 29.4805 | -6.98052 | -15.47953184 | 1.518491844 | -0.382147147 |

**Supplementary Table 3C**

**Coil-mIFX in naïve BALB/c mice and immunized BALB/c mice**

| **Time (hr)** | ***P* value** | **Mean1** | **Mean2** | **Difference** | **CI Lower** | **CI Upper** | **Effect size**  **(Cohen’s d)** |
| --- | --- | --- | --- | --- | --- | --- | --- |
| 0 | >0.9999 | 0 | 0 | 0 | -14.20523684 | 14.20523684 | 0 |
| 0.5 | 0.612388 | 10.1867 | 6.60667 | 3.58 | -10.62523684 | 17.78523684 | 0.117258903 |
| 4 | 0.00433538 | 10.3067 | 31.6333 | -21.3267 | -35.53193684 | -7.121463156 | -0.698532249 |
| 24 | <0.0001 | 9.12 | 43.0067 | -33.8867 | -48.09193684 | -19.68146316 | -1.109921026 |
| 72 | 0.00429209 | 13.3267 | 34.68 | -21.3533 | -35.55853684 | -7.148063156 | -0.699403502 |
| 144 | 0.95101 | 15.04 | 14.6067 | 0.433336 | -13.77190084 | 14.63857284 | 0.014193437 |

**Supplementary Table 4A**

**hTNFα vs. Infliximab+ hTNFα (hTNFα concentration)**

| **Time (min)** | ***P* value** | **Mean1** | **Mean2** | **Difference** | **CI Lower** | **CI Upper** | **Effect size**  **(Cohen’s d)** |
| --- | --- | --- | --- | --- | --- | --- | --- |
| 10 | 0.0138276 | 5169.93 | 4017.83 | 1152.1 | 388.3332689 | 1915.866731 | 2.418008947 |
| 60 | 0.00018703 | 3817.17 | 557.133 | 3260.03 | 2577.338242 | 3942.721758 | 7.654651112 |

**Supplementary Table 4B**

**hTNFα vs. Pro-Infliximab+ hTNFα (hTNFα concentration)**

| **Time (min)** | ***P* value** | **Mean1** | **Mean2** | **Difference** | **CI Lower** | **CI Upper** | **Effect size**  **(Cohen’s d)** |
| --- | --- | --- | --- | --- | --- | --- | --- |
| 10 | 0.261035 | 5169.93 | 4726.6 | 443.333 | -497.8513498 | 1384.51735 | 0.755064108 |
| 60 | 0.996514 | 3817.17 | 3818.57 | -1.3999 | -837.6901537 | 834.8903537 | -0.002683295 |
